# Calcium regulates acid-sensing ion channel 3 activation by competing with protons in the channel pore and at an allosteric binding site

**DOI:** 10.1098/rsob.220243

**Published:** 2022-12-21

**Authors:** Sophie Roy, Niklaus Johner, Viktor Trendafilov, Ivan Gautschi, Olivier Bignucolo, Ophélie Molton, Simon Bernèche, Stephan Kellenberger

**Affiliations:** ^1^ Department of biomedical Sciences, University of Lausanne, 1011 Lausanne, Switzerland; ^2^ Swiss Institute of Bioinformatics, 1015 Lausanne, Switzerland; ^3^ Biozentrum, University of Basel, 4056 Basel, Switzerland

**Keywords:** ASIC, ion channel, calcium, activation, pH dependence, molecular dynamics

## Abstract

The extracellular Ca^2+^ concentration changes locally under certain physiological and pathological conditions. Such variations affect the function of ion channels of the nervous system and consequently also neuronal signalling. We investigated here the mechanisms by which Ca^2+^ controls the activity of acid-sensing ion channel (ASIC) 3. ASICs are neuronal, H^+^-gated Na^+^ channels involved in several physiological and pathological processes, including the expression of fear, learning, pain sensation and neurodegeneration after ischaemic stroke. It was previously shown that Ca^2+^ negatively modulates the ASIC pH dependence. While protons are default activators of ASIC3, this channel can also be activated at pH7.4 by the removal of the extracellular Ca^2+^. Two previous studies concluded that low pH opens ASIC3 by displacing Ca^2+^ ions that block the channel pore at physiological pH. We show here that an acidic residue, distant from the pore, together with pore residues, controls the modulation of ASIC3 by Ca^2+^. Our study identifies a new regulatory site in ASIC3 and demonstrates that ASIC3 activation involves an allosteric mechanism together with Ca^2+^ unbinding from the channel pore. We provide a molecular analysis of a regulatory mechanism found in many ion channels.

## Graphical abstract

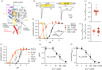

## 1. Introduction

Intracellular Ca^2+^ is an established secondary messenger. The regulatory role of extracellular Ca^2+^ is less widely known. Extracellular free Ca^2+^ concentrations are locally decreased during high neuronal activity or seizures and in ischaemia [1–3]. These changes can have a strong effect on neuronal excitability [4], since the extracellular Ca^2+^ concentration affects the properties of many neuronal ion channels [5–8]. In the present study, we investigated the molecular mechanism by which the activity of one such Ca^2+^-sensitive channel, the acid-sensing ion channel (ASIC) 3, is controlled by extracellular Ca^2+^. ASICs are H^+^-gated, Na^+^-conducting ion channels of the nervous system [9–11]. Extracellular acidification leads to a rapid activation of the ASIC current. This current is transient in the continued presence of a low pH solution, because the channels enter a non-conducting, desensitized state after opening [9,10,12]. Functional ASIC channels are formed by the trimeric assembly of identical or homologous subunits. In rodents, six ASIC subtypes encoded by four genes are known [9,10]. Of these, ASIC1a contributes the high pH sensitivity of ASICs of the CNS [13,14], while ASIC3 is the most important ASIC subtype in the PNS [15].

**Figure 1.**
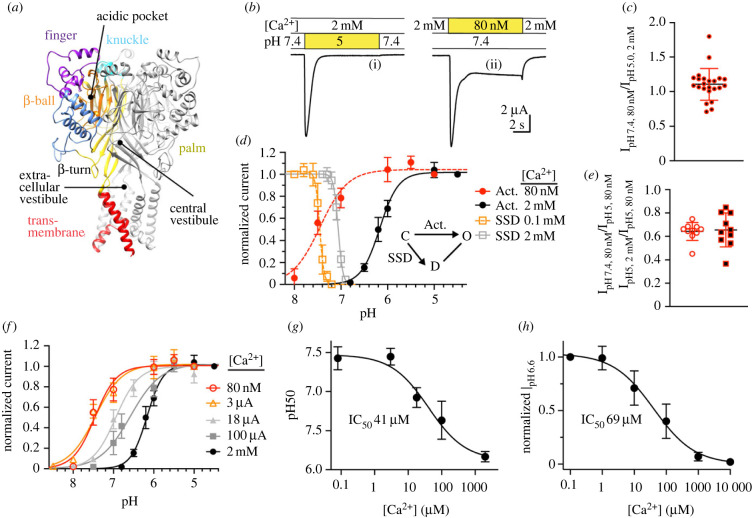
Competition between H^+^ and Ca^2+^ on ASIC3. (*a*) Structural image of an ASIC3 trimer, showing one subunit with domain-specific colouring and the other two subunits in grey. The domains are labelled in the coloured subunit. The ASIC3 model is based on the open structure of chicken ASIC1a [17]. (*b–h*) Data are from *Xenopus* oocytes expressing ASIC3 WT, obtained by two-electrode voltage clamp to −60 mV. The conditioning solution at pH7.4 (= the solution perfused between stimulations) contained 2 mM Ca^2+^ except where noted; the Ca^2+^ concentration in the stimulation solutions was as indicated. (*b*) Representative current traces showing ASIC3 activation by lowering of the pH (i) and by lowering of the Ca^2+^ concentration (ii). (*c*) Ratio of the current induced by the two approaches described in (*b*). Ratios were calculated in individual cells, *n* = 13. (*d*) pH dependence of activation (filled circles) and of SSD (open squares). The protocols are described in the methods. The Ca^2+^ concentration was generally 2 mM. For the activation curves, the (free) Ca^2+^ concentration of the stimulation was as indicated in the figure; for SSD curves, the Ca^2+^ concentration of the conditioning solution was as indicated in the figure; *n* = 12–16. The inset shows a kinetic scheme of ASIC3 with the three functional states closed (C), open (O) and desensitized (D). The transitions corresponding to activation and SSD are indicated by arrows. (*e*) Current ratios measured in individual cells, of *I*_pH7.4_/*I*_pH5.0_ obtained at 80 nM free Ca^2+^ (red circles), and of the I_2mM_/I_80nM_ Ca^2+^ at pH5 (red-black squares), *n* = 10. (*f*) Activation pH dependence measured at different free Ca^2+^ concentration in the stimulation solution, as indicated; *n* = 8–14. (*g*) Plot of the pH_50_ as a function of the free Ca^2+^ concentration in the stimulation solution, based on (*f*). (*h*) Inhibition of pH6.6-induced current by Ca^2+^. The conditioning pH was 7.4 at a Ca^2+^ concentration of 2 mM. Channel activation was induced by changing to a solution at pH6.6 at the indicated free Ca^2+^ concentration for 10 s once every min, *n* = 10.

The shape of an ASIC subunit is comparable to that of a hand holding a small ball, with the forearm being the transmembrane segment; the extracellular domains have accordingly been named as finger, knuckle, palm and thumb [16] (figure 1*a*). The ectodomain is composed of a central scaffold made up by the palm and the knuckle. The thumb and finger contain several *α* helices and are oriented towards the outside of the protein. The ASIC trimer contains two vestibules located along the central vertical axis between the three subunits, the ‘extracellular vestibule’, situated directly above the pore entry, and the ‘central vestibule’, enclosed by the *β* sheets of the lower palm domains (figure 1*a*). The ‘acidic pocket’, present three times in the trimer, is located at about 40 Å above the pore, enclosed by the finger, thumb and β-ball domains of one subunit, and the palm of a neighbouring subunit [16–19].

The ASIC pH dependence is inversely correlated with the extracellular Ca^2+^ concentration, suggesting that there is a competition between H^+^ and Ca^2+^ ions for common binding sites [8,20]. Changing of the extracellular medium at pH7.4 to a nominally Ca^2+^-free solution at the same pH induced an inward current in ASIC3 [8,21]. Based on the dependence of the macroscopic currents on the Ca^2+^ and H^+^ concentrations and the observation that unitary current amplitudes were decreased by high Ca^2+^ concentrations, it was concluded that acidification activates ASIC3 by a displacement of Ca^2+^ ions bound to the pore entry at neutral and alkaline pH, without conformational changes [8]. A recent study with ASIC3 showed that if the residue Glu435 in the pore region of ASIC3, which is not conserved in ASIC1a, was mutated, the current induced by Ca^2+^ removal at pH7.4 was strongly decreased, further supporting the hypothesis that the competition between H^+^ and Ca^2+^ occurs in the ASIC3 pore. The E435A mutation did, however, only partially suppress the low Ca^2+^-induced current. In the present work, we used several complementary approaches to determine the contribution of sites in and outside the ASIC3 pore to the gating control by Ca^2+^. We show that in addition to the previously identified Glu435 in the pore entry, a residue in the acidic pocket and an acidic residue deeper down in the channel pore contribute to the control of ASIC activation by Ca^2+^. Protons activate ASIC3 therefore by binding to H^+^-sensing residues that lead, via conformational changes, to the opening of the pore and by displacing Ca^2+^ bound into the pore entry.

## 2. Results

### 2.1. Lowering of the Ca^2+^ concentration shifts the pH dependence of acid-sensing ion channel 3

When ASIC3 was expressed in *Xenopus laevis* oocytes and its function was measured by two-electrode voltage clamp, extracellular acidification to pH5 resulted in large, transient inward currents (figure 1*b*i). If the pH was kept at 7.4, and the free Ca^2+^ concentration was lowered from 2 mM to 80 nM, a transient current of similar amplitude as that induced by the acidification was measured (figure 1*b*ii; measurement from the same oocyte), as shown previously [8,21]. In this series of experiments, the peak current amplitudes induced with pH5/2 mM Ca^2+^ and pH7.4/80 nM Ca^2+^ were roughly equal (figure 1*c*). *Xenopus* oocytes express endogenous connexin hemichannels that are activated by lowering of the extracellular Ca^2+^ concentration [22]. Control experiments showed that under our measuring conditions, the endogenous low Ca^2+^-activated currents had a much smaller amplitude (less than or equal to 93 ± 50 nA, *n* = 29) and slower kinetics than the ASIC currents and did therefore not interfere with the ASIC current measurements (see Methods; electronic supplementary material, figure S1). To examine whether the low Ca^2+^-induced current can be explained by a changed pH sensitivity of ASIC3, the pH dependence of activation was determined at the two Ca^2+^ concentrations 2 mM and 80 nM. The pH dependence of ASIC activation was measured by stimulating the channels with a series of solutions of increasingly acidic pH. Fitting the pH dependence curves obtained at 80 nM free Ca^2+^ and at 2 mM Ca^2+^ yielded pH values of half-maximal activation (pH_50_) of 7.48 ± 0.14 (*n* = 62) and 6.14 ± 0.11 (*n* = 42), respectively, for the two Ca^2+^ concentrations, confirming therefore a strong alkaline shift of the ASIC3 pH dependence upon lowering of the Ca^2+^ concentration (figure 1*d*). According to the activation curve, pH7.4 induces in the 80 nM Ca^2+^ condition 64 ± 0.8% of the maximal current achieved at this Ca^2+^ concentration (*n* = 10, figure 1*e*, red circles). By contrast, in the 2 mM Ca^2+^ condition, no current was measured at pH7.4 (figure 1*d*). In addition to its effects on pH dependence, Ca^2+^ can, at millimolar concentrations, inhibit ASICs by a pore block mechanism [8,23]. When pH5-induced current amplitudes were compared between the two Ca^2+^ concentrations, the current ratio *I*pH5(2 mM Ca^2+^)/*I*pH5 (80 nM Ca^2+^) was 0.65 ± 0.15 (*n* = 10, figure 1*e*, red-black squares), indicating that there is an approximately 35% inhibition of the maximal peak current amplitude at pH5 by 2 mM Ca^2+^. The similar amplitudes of the low Ca^2+^(pH7.4/80 nM Ca^2+^)- and low pH (pH5/2 mM Ca^2+^)-induced currents (figure 1*b,c*) are therefore due (i) to the shift in pH dependence of activation leading to an increase of the current amplitude in the low Ca^2+^ condition of approximately 65% of the maximal amplitude (figure 1*d*) and (ii) to the 35% pore block by 2 mM Ca^2+^ at pH5. Lowering of the extracellular Ca^2+^ concentration also shifts the pH dependence of the transition from the closed to the desensitized state, termed steady-state desensitization (SSD), to more alkaline values (SSD in the inset of figure 1*d*). To determine the pH dependence of SSD, ASIC3-expressing oocytes were perfused with conditioning pH solution for 55 s, before the fraction of not-desensitized channels was measured by a step to pH5; this protocol was repeated with increasingly acidic conditioning solutions. In these experiments, the Ca^2+^ concentration in the conditioning solution was 2 mM in one series, and 0.1 mM in the second series, while the pH of the stimulation solution was 5, and it contained in both series 2 mM Ca^2+^. The lowering of the Ca^2+^ concentration in the conditioning solution induced a substantial alkaline shift of the pH dependence of SSD. In the 2 mM Ca^2+^ condition, the SSD and activation curves did not overlap, while there was a large overlap in the low Ca^2+^ condition (figure 1*d*). To avoid channel activation by the conditioning solution, a concentration of 0.1 mM had been chosen for the low Ca^2+^ condition of the SSD, thus much higher than the 80 nM of the activation experiments. At a Ca^2+^ concentration of 80 nM this curve would be shifted to more alkaline values, and the overlap would be smaller. To estimate the affinity of the Ca^2+^ binding site, activation curves at different free Ca^2+^ concentrations were recorded (figure 1*f*). The plot of the pH_50_ values as a function of the Ca^2+^ concentration (figure 1*g*) indicates an IC_50_ of 41 µM. As another measure of Ca^2+^ binding affinity, the ASIC3 current was determined at pH6.6 at different free Ca^2+^ concentrations, showing smaller current amplitudes with increasing Ca^2+^ concentration. The IC_50_ measured under these conditions was 69 ± 52 µM (*n* = 11, figure 1*h*). This IC_50_ depends on the pH, since there is a competition between Ca^2+^ and H^+^. At pH 7.0 the Ca^2+^ IC_50_ was 6.9 ± 6.8 µM (*n* = 11, electronic supplementary material, figure S2).

### 2.2. A chimera approach identifies channel domains important for the Ca^2+^ sensitivity

**Figure 2.**
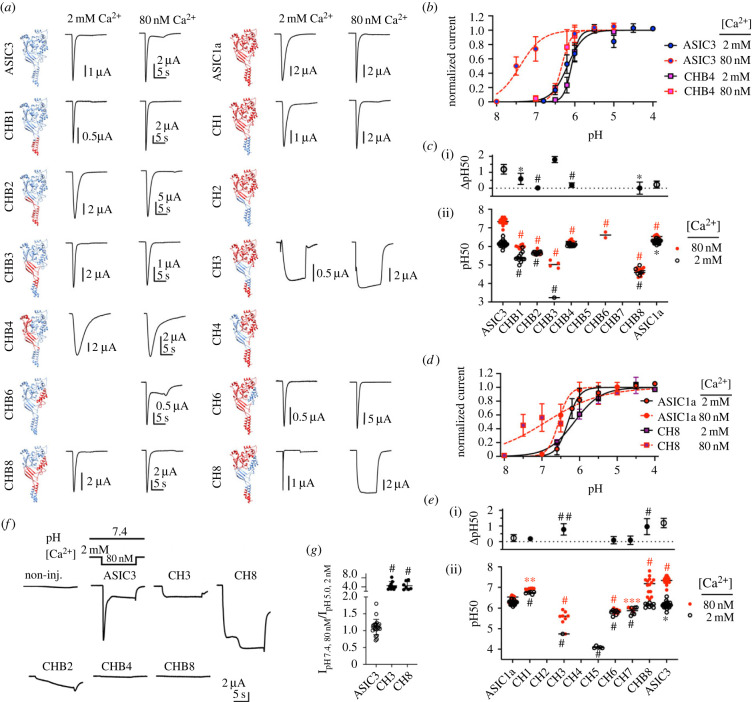
ASIC3-ASIC1a chimeras identify channel domains important for Ca^2+^ sensitivity. Data are from *Xenopus* oocytes expressing the indicated ASIC constructs, obtained by two-electrode voltage clamp to −60 mV. (*a*) Schematic view and representative current traces of chimeras, induced by a pH close to the pH_50_. Left column, chimeras based on ASIC3, in which the parts highlighted in red (table 1) were replaced by the corresponding ASIC1a sequence. On the right of the structural images, representative current traces measured in a stimulation solution containing 2 mM or 80 nM free Ca^2+^ are shown. Right column, chimeras based on ASIC1a, in which the parts highlighted in blue (table 1) were replaced by the corresponding ASIC3 sequence; representative current traces on the right. Empty fields indicate that for a given chimera/condition no current was recorded. (*b*) Activation curves of ASIC3 WT and the chimera CHB4; *n* = 10–15. (*c*) (ii), pH_50_ values obtained at 2 mM and 80 nM free Ca^2+^ in the stimulation solution for ASIC1a, ASIC3 and the indicated ASIC3-based chimeras; *n* = 2–21; (i), the *Δ*pH_50_ (pH_50,80nM_ - pH_50,2mM_ Ca^2+^, mean ± s.d.) values are plotted for the indicated constructs. CHB3 and CH3 (in (*e*)) showed with 2 mM Ca^2+^ irreversible rundown at more acidic pH. Their complete pH dependence is therefore assembled from data from different cells, and one single pH_50_ from the pooled data was determined. (*d*) Activation curves of ASIC1a WT and the chimera CH8; *n* = 8–11. (*e*) (ii), pH_50_ values obtained at 2 mM and 80 nM free Ca^2+^ in the stimulation solution for ASIC1a, ASIC3 and the indicated ASIC1a-based chimeras; *n* = 5–24. (i), the *Δ*pH_50_ (pH_50,80nM_ - pH_50,2mM_ Ca^2+^, mean ± s.d). Values are plotted for the indicated constructs. (*f*) Representative current traces of oocytes expressing the indicated constructs, in response to a lowering of the extracellular Ca^2+^ concentration to 80 nM at pH7.4. (*g*) Ratio of the current induced by lowering the Ca^2+^ concentration at pH7.4 from 2 mM to 80 nM / current induced by acidification from pH7.4 to pH5.0 at 2 mM Ca^2+^, *n* = 7–23. (*c,e,g*) *, *p* < 0.05; **, *p* < 0.01; ***, *p* < 0.001; ^#^, *p* < 0.0001; ^##^, *p* = 0.089; different from the corresponding value obtained with WT ASIC1a or WT ASIC3; (*g*) and (ii) of (*c*) and (*e*), as analysed by one-way ANOVA followed by Dunnett's test; (i) of (*c*) and (*e*), based on permutation analysis (see Methods).

The observed shift of pH activation curves by changes of the Ca^2+^ concentration is likely due to a competition for binding sites between H^+^ and Ca^2+^ ions. The identification of the amino acid residues of ASIC3 to which Ca^2+^ binds would indicate where the competition occurs. As a first approach towards this aim, subdomain-based chimeras between ASIC3 and ASIC1a, which is much less Ca^2+^-sensitive, were constructed (figure 2, table 1). Constructs named ‘CHB#’ (where ‘#’ is a number) are based on ASIC3 (blue in figure 2*a*, left column), and the subdomains indicated in table 1 are replaced by the corresponding sequence of ASIC1a (red in figure 2*a*). The inverse chimeras (ASIC3 in ASIC1a) are named ‘CH#’. Most of the constructed chimeras were functional and expressed transient currents (figure 2*a*; electronic supplementary material, figure S3). The pH_50_ values of ASIC1a and ASIC3 WT are quite similar at 2 mM Ca^2+^ but different at 80 nM Ca^2+^ (figure 2*c*ii,*e*ii). Lowering the Ca^2+^ concentration from 2 mM to 80 nM Ca^2+^ induced an alkaline shift of the pH_50_ values (ΔpH_50_) of 1.19 ± 0.30 and 0.23 ± 0.22 (mean ± sum of s.d.; *n* = 12–21), respectively, in ASIC3 and ASIC1a (figure 2*c*i). Of the ASIC1a-in-ASIC3 chimeras, CHB1, CHB2, CHB4 and CHB8 decreased the Ca^2+^-induced ΔpH_50_ (figure 2*b,c*), suggesting a possible role of the ASIC1a intracellular and transmembrane segments and lower palm (CHB1, CHB2), the palm and knuckle (CHB4), and the upper transmembrane and lower palm parts together with the palm-thumb loops and the thumb (CHB8) in reducing this Ca^2+^ sensitivity. Of the ASIC3-in-ASIC1a chimeras, CH8 showed an increased, and CH3 showed a tendency of increased *Δ*pH_50_ (*p* = 0.089) relative to ASIC1a WT (figure 2*d,e*). CH3 contains the upper transmembrane and lower palm parts of ASIC3, whereas CH8 contains the ASIC3 upper transmembrane and lower palm parts together with the palm-thumb loops and the thumb. The currents at 80 nM Ca^2+^ of both CH3 and CH8 were sustained. These currents activated rapidly upon the pH change; their kinetics and amplitude were thus different from the endogenous currents (electronic supplementary material, figure S1). The disruption of the desensitization in these chimeras may be due to the sequence changes in the palm, which controls desensitization. For selected chimeras, it was tested whether lowering of the Ca^2+^ concentration from 2 mM to 80 nM free Ca^2+^ at pH7.4 induced a current. Figure 2*f* compares representative current traces of these chimeras to those of non-injected or ASIC3 WT-expressing oocytes. Of the ASIC3-based chimeras, CHB2 generated a slowly increasing current, while the current of CHB4 and CHB8 was not different from that of non-injected oocytes, indicating that in these two chimeras, the activation by low Ca^2+^ was disrupted. The low Ca^2+^-induced current, normalized to the low pH-induced current in the same oocyte (the *I*pH7.4(80 nM Ca^2+^) / *I*pH5(2 mM Ca^2+^) ratio) was high for CH3 and CH8, as expected, even higher than in ASIC3 (figure 2*g*). Taken together, the chimera analysis highlights the importance of the pore and pore entry for the gating by Ca^2+^, as well as that of the palm, thumb and knuckle.

**Table 1.** Chimera constructs.

Name	introduced subdomains
CH(B)1	intracellular termini, transmembrane part
CH(B)2	lower palm, transmembrane part
CH(B)3	upper transmembrane, lower palm, palm side of palm-thumb linkers
CH(B)4	palm, knuckle
CH(B)5	β-ball
CH(B)6	thumb, thumb part of palm-thumb linkers
CH(B)7	finger
CH(B)8	upper transmembrane part, lower palm, palm-thumb linkers, thumb
CHx	the listed domains of ASIC3 were introduced in ASIC1a; CHBx, the listed domains of ASIC1a were introduced in ASIC3

### 2.3. MD simulations predict candidate Ca^2+^ binding sites

**Figure 3.**
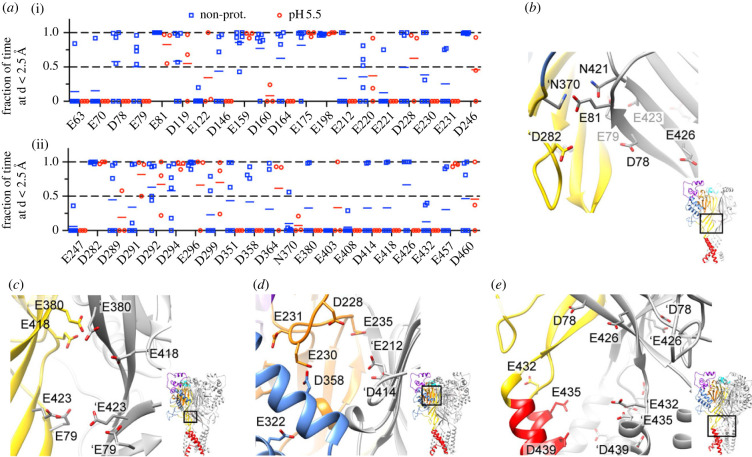
Molecular dynamics simulations predict candidate Ca^2+^ binding sites. (*a*) Plot of the fraction of time, a Ca^2+^ ion is closer than 2.5 Å from the indicated residue. Simulations were carried out without the protonation of side chains (blue squares) or at the protonation of side chains estimated at pH5.5 (red circles, using the program propKa; see *Methods;* values are from 3 to 6 subunits). (*b–e*) structural images made from a model of ASIC3, based on the open structure of chicken ASIC1a [17]. Focused views of specific regions. One subunit is shown with domain-specific colouring, the other two subunits are shown in grey. (*b*) Lower palm with intersubunit interaction site above the β-turn; (*c*) Central vestibule enclosed by the lower palm β-sheets; (*d*) acidic pocket; (*e*) lower palm and upper part of transmembrane domains. Residues identified in (*a*) as potentially involved in Ca^2+^ binding are shown in (*b*–*e*).

To identify candidate Ca^2+^ binding sites, MD simulations with a structural model of ASIC3 were carried out in the presence of a high concentration of Ca^2+^ (100 mM). Analyses were performed to identify the Glu, Asp, Asn, His and Thr amino acid residues of ASIC3, in whose proximity (i.e. at a distance of less than 2.5 Å), a Ca^2+^ ion was found during a part of the simulation. Figure 3*a* plots the fraction of time a Ca^2+^ was close to the indicated residues of the transmembrane and extracellular parts of ASIC3 in two conditions, either without protonation of acidic side chains (Blue squares), or with a chosen protonation corresponding approximately to pH5.5 (red circles, *Methods*). We expect that acidification displaces Ca^2+^ from sites where Ca^2+^ and H^+^ compete, thus that at such sites, Ca^2+^ would not be found in the pH5.5 condition. In MD simulations, Ca^2+^ ions tend to stick to a site once they have bound. Residues to which Ca^2+^ bound in most of the experiments are therefore more likely than others to bind Ca^2+^. The computational analysis led together with inspection of the ASIC3 structural model to the identification of several clusters of residues in whose proximity Ca^2+^ ions were found. In the lower palm domain, at a subunit interaction site, Glu81, which had during most of the simulation time a Ca^2+^ ion bound, is close to Asn421 of the same subunit, and to ′Asn370 of a neighbouring subunit (figure 3*b*; residue names of the neighbouring subunit are preceded by the prefix’). ′Asp282, located further down just above the β-turn, had also a Ca^2+^ ion bound during most of the simulation time. Asp78 and Glu426 that also scored high are farther away. Glu79 and Glu423 of the lower palm are oriented towards the inside of the central vestibule where these residues of the three subunits may form a Ca^2+^ binding site (figure 3*c*). Glu423 did not score in our analysis but may be of interest because it has been suggested to bind 2-guanidine-4-methylquinazoline, which is known to compete with Ca^2+^ for binding sites [24,25]. Further up in the central vestibule, Glu380 and Glu418, identified in our screening, may form an additional binding site in the central cavity. Several residues were identified in our screening that are part of the acidic pocket, which has been identified as Ca^2+^ binding site in ASIC1a [26] (figure 3*d*). Of these, ′Glu212, Asp228, Glu230 and Asp358 all had frequently a Ca^2+^ in their proximity (figure 3*a*). The structural image shows that Glu230 and Asp358 are oriented towards each other, while ′Glu212 of a neighbouring subunit is oriented towards Glu235. ′Asp414 has a similar orientation as ′Glu212 and is located on an adjacent, antiparallel strand of the same palm β-sheet (figure 3*d*). A potentially interesting cluster was identified at the extracellular pore entry (figure 3*e*). Glu432 and Asp439 are homologous to the two Ca^2+^ pore-blocking binding residues identified in ASIC1a [23]. Glu435 is unique to ASIC3; ASIC1a contains a Gly residue at the homologous position. Some residues that scored in our analysis appeared not to be of interest after inspection of the structural model since they are located in a loop region that is not well conserved between ASIC3 and ASIC1a, on which the structural model is based (electronic supplementary material, figure S4A), or they appeared to be too distant from other acidic residues (electronic supplementary material, figure S4B,C).

### 2.4. Functional analysis of mutants identifies the pore entry and the acidic pocket as likely Ca^2+^ binding sites for activation

**Figure 4.**
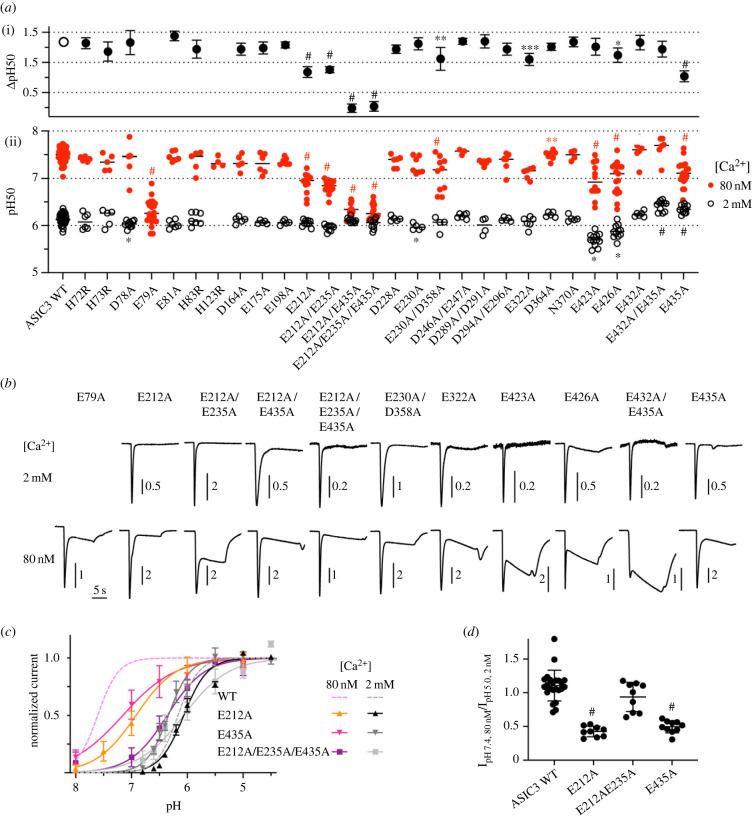
Functional analysis of mutants identifies the pore entry and the acidic pocket as likely Ca^2+^ binding sites for activation. Data are from *Xenopus* oocytes expressing the indicated ASIC3 mutants, obtained by two-electrode voltage clamp to −60 mV. (*a*) (ii), for the indicated mutants, pH_50_ values obtained from activation curves are plotted for conditions with stimulation solutions containing 80 nM or 2 mM free Ca^2+^, *n* = 3–63; (i), the *Δ*pH_50_ (pH_50,80nM_ – pH_50,2mM_ Ca^2+^, mean ± s.d.) values are plotted for the indicated constructs. (*b*) Representative current traces of mutants showing a difference in the pH dependence relative to WT, obtained at 80 nM or 2 mM Ca^2+^, at a pH close to the pH_50_. (*c*) Activation curves of the indicated mutants, *n* = 8–27. For comparison, the pH dependence of ASIC3 WT is shown as dashed lines (80 nM, purple, 2 mM, grey). (*d*) Ratio of the current induced by lowering the Ca^2+^ concentration at pH7.4 from 2 mM to 80 nM / current induced by acidification from pH7.4 to pH5.0 at 2 mM Ca^2+^, *n* = 9–23. *, *p* < 0.05; **, *p* < 0.01; ***, *p* < 0.001; ^#^, *p* < 0.0001; different from the corresponding value obtained with WT ASIC3; (*d*) and (ii) of (*a*), as analysed by one-way ANOVA followed by Dunnett's test; (i) of (*a*), based on permutation analysis (see Methods).

Based on the predictions of the MD simulations and of visual inspection of the ASIC3 structural model, approximately 30 residues—most of them acidic residues—were mutated to Ala, and the pH dependence of activation was determined at the two Ca^2+^ concentrations. Representative current traces of mutants that showed a difference to WT ASIC3 are shown at the two Ca^2+^ concentrations, at a pH close to the pH_50_ (figure 4*b*). The pH_50_ values measured at 2 mM Ca^2+^ were remarkably constant across mutants (black open circles in the figure 4*a*ii). The largest shifts were observed with the mutations E423A and E426A. E79A produced only a very small current in these conditions, precluding the analysis of the pH dependence. The pH_50_ obtained at 80 nM free Ca^2+^ was more affected by the mutations. Mutation of Glu212, located in the palm and facing the acidic pocket (figure 3*d*), induced an acidic shift of the pH_50_ (figure 4*a,b*) and decreased the ΔpH_50_ to 0.84 ± 0.29 (*n* = 9–12, figure 4*a*i), compared to 1.34 ± 0.25 in ASIC3 WT (*n* = 42–62). Mutation of the close residue Asp414 resulted in non-functional channels. If the ′E212A mutation was combined with the mutation of Glu235, which is oriented towards ′Glu212, the *Δ*pH_50_ was not further decreased (figure 4*a*). The E435A mutation (figure 3*e*), which was previously shown to decrease the low Ca^2+^-induced current [21], lowered the ΔpH_50_ value to 0.77 ± 0.35 (figure 4*a,c*, *n* = 11–19), thus similarly to E212A. In ASIC1a, the residues corresponding to Glu432 and Asp439 were identified as binding sites for pore block by Ca^2+^ [23]. In the E432A mutant, the *Δ*pH_50_ was similar to the WT value (figure 4*a*). The D439A mutant did not produce any transient currents. Combination of the mutations of Glu435 of the pore and of Glu212 of the palm, with or without mutation of Glu235, resulted in a strong reduction of the pH_50_ shift upon lowering of the Ca^2+^ concentration, with *Δ*pH_50_ values of 0.24 ± 0.20 (E212A/E435A, *n* = 9–10) and 0.27 ± 0.27 (E212A/E235A/E435A, *n* = 9–14; figure 4*a,c*). Comparison with the WT pH dependence (dashed lines in figure 4*c*) shows that these mutations affected mostly the channel properties in the 80 nM Ca^2+^ condition, and much less in the 2 mM Ca^2+^ condition. Other mutants with a significantly lower ΔpH_50_ than ASIC3 WT, but with smaller effects, were E230A/D358A, E322A and E426A (figure 4*a,b*). E230 and D358 are located in the acidic pocket (figure 3*d*), E322 just below, in the *α*4 helix of the thumb, while E426 is in the wrist (figure 3*b*). Measurement of the low Ca^2+^-induced current at pH7.4 demonstrated a decrease of the *I*pH7.4(80 nM Ca^2+^) / *I*pH5(2 mM Ca^2+^) ratio by the single mutations (figure 4*d*), confirming further the importance of these two sites for modulation by Ca^2+^. The low Ca^2+^-induced currents of the mutant E212A/E435A showed a slowly developing sustained current without a clear peak that could not be reliably quantified. The mutational analysis indicates therefore that two distinct Ca^2+^ binding sites, one in the pore entry and one in the palm/acidic pocket, contribute to the Ca^2+^ modulation of ASIC3 activation, consistent with the analysis of the chimeras.

### 2.5. Acid-sensing ion channel 3 concatamers highlight the importance of the pore residue Asp439 for modulation by Ca^2+^

**Figure 5.**
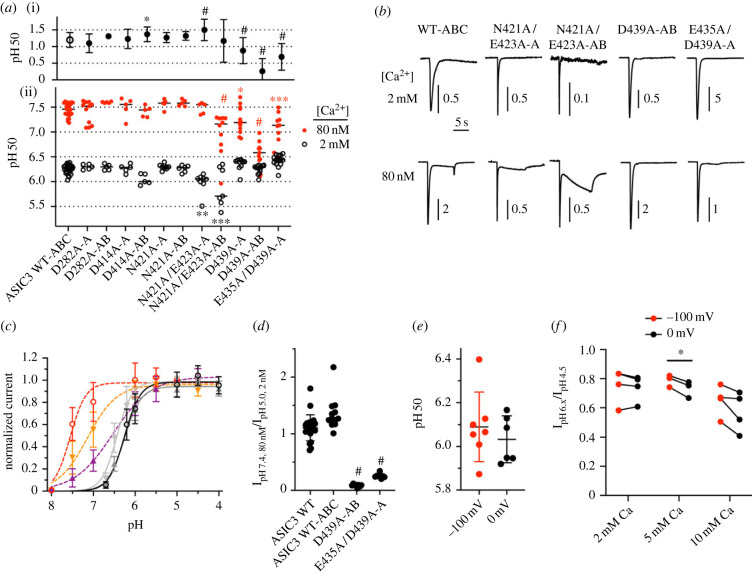
ASIC3 concatamers highlight the importance of the pore residue D439 for modulation by Ca^2+^. Data are from *Xenopus* oocytes expressing the indicated ASIC constructs, obtained by two-electrode voltage clamp, to −60 mV (*a*–*d*) or as indicated (*e*,*f*). (*a*) (ii), for the indicated mutants, pH_50_ values obtained from activation curves are plotted for conditions with stimulation solutions containing 80 nM or 2 mM free Ca^2+^, *n* = 4–42. The letters at the end of the names of the mutants (A, AB) indicate in which of the subunits A, B, C the mutation was present in a given construct; (i), the *Δ*pH_50_ (pH_50,80nM_ - pH_50,2mM_ Ca^2+^, mean ± s.d.) values are plotted for the indicated constructs; (*b*) representative current traces from mutants showing a difference in the pH dependence relative to the WT concatamer, obtained at 80 nM or 2 mM Ca^2+^, at a pH close to the pH_50_. (*c*) Activation curves of the indicated mutants, with the WT concatamer for comparison, *n* = 9–20. (*d*) Ratio of the current induced by lowering the Ca^2+^ concentration at pH7.4 from 2 mM to 80 nM / current induced by acidification from pH7.4 to pH5.0 at 2 mM Ca^2+^, *n* = 8–23. (*e,f*) Voltage dependence of ASIC3 WT modulation by Ca^2+^. Currents were measured at the indicated voltage. (*a–d*) *, *p* < 0.05; **, *p* < 0.01; ***, *p* < 0.001; ^#^, *p* < 0.0001; different from the corresponding value obtained with ASIC3 WT-ABC; (*d*) and (ii) of (*a*), as analysed by one-way ANOVA followed by Dunnett's test; (i) of (*a*), based on permutation analysis (see Methods). (*e*) pH_50_ values determined from full activation curves in extracellular solutions containing 2 mM Ca^2+^, carried out at −100 or 0 mV, as indicated, *n* = 6–7. (*f*) Current ratios between current induced by a pH located in the steep range of the activation curve / *I*pH4.5, at the indicated Ca^2+^ concentration and voltage, *n* = 3–4. pH6.x was 6.2 at 2 mM Ca^2+^, 6.1 at 5 mM Ca^2+^ and 6.0 at 10 mM Ca^2+^. *, *p* < 0.05, different between the two potentials.

Several mutations resulted in channels that did not express transient currents, among them mutations of Asp439, the residue homologous to a pore-blocking site in ASIC1a. To allow the study of such sites, concatamers were constructed in which residues of interest were mutated only in one or two subunits of the channel trimer. All tested concatamers carrying mutations in one or two subunits, except the one containing the double mutation E435A/D439A in two subunits of the trimer, produced measurable, transient currents. The pH dependence of concatamers carrying the mutations D282A or N421A of the wrist (figure 3*e*) or D414A of the acidic pocket (figure 3*d*; ‘A’, ‘AB’ at the end of the name indicates in which subunits of the trimer [A, B, C] a given mutation is present) in one or two of the subunits was not different from that of WT (figure 5*a*), thus it is unclear why the conventional mutants D282A, N421A and D414A, carrying these mutations in the three subunits, were not functional. The analysis of the concatamers provided the most interesting results in the pore region, where the presence of the mutation D439A or the double mutation E435A/D439A in one subunit of the trimer led to a ΔpH_50_ of 0.88 ± 0.39 (*n* = 11–12, D439A-A) and 0.69 ± 0.40 (*n* = 9–14, E435A/D439A-A), respectively, compared to 1.20 ± 0.22 of the WT concatamer ABC (figure 5*a–c*; *n* = 25–26). In the construct containing the D439A mutation in two of the three subunits, the *Δ*pH_50_ was 0.26 ± 0.38 (figure 5*a*, *n* = 12–13), highlighting the importance of Asp439 for the Ca^2+^ modulation of the ASIC3 pH dependence. Representative current traces of concatamers with different activation pH dependence from WT are shown in figure 5*b*. Consistent with the above observations, the *I*pH7.4(80 nM Ca^2+^) / *I*pH5(2 mM Ca^2+^) ratio was low in the concatamers E435A/D439A-A and D439A-AB (figure 5*d*). This indicates that residues Glu435 and Asp439 contribute together to the Ca^2+^ binding site in the pore entry.

To test whether the modulation of the pH dependence by Ca^2+^ depended on the membrane potential, complete pH dependence curves were carried out with ASIC3 WT at either −100 or 0 mV. The pH_50_ values obtained were not different between these two conditions (figure 5*e*). To have a more direct comparison than activation curves obtained in different cells, the ratio of the current induced by a pH of the steep range of the pH-current curve, divided by the current induced by a pH generating maximal activation, was measured at −100 and 0 mV at different Ca^2+^ concentrations in paired experiments in the same cell (figure 5*f*). This *I*_pH6.x_/*I*_pH4.5_ ratio is sensitive to changes in the pH dependence. If Ca^2+^ binding to sites in the electrical field of the pore competes with pH-dependent activation, the *I*_pH6.x_/*I*_pH4.5_ ratio would be lower at the membrane potential of −100 mV. In contrast with these expectations, the *I*_pH6.x_/*I*_pH4.5_ ratio was significantly lower at 0 mV in the condition with 5 mM Ca^2+^ and showed no voltage dependence with 2 and 10 mM Ca^2+^ (figure 5*f*). This suggests that the Ca^2+^ binding site at residues Glu435 and Asp439 is located outside the electrical field of the pore, unlike the binding site of diminazene, which comprises residues corresponding to Gly442, Gly445 and Leu446 in ASIC3, and is closer to the selectivity filter [27,28].

### 2.6. The Ca^2+^ modulation of steady-state desensitization depends on residues in the acidic pocket and the lower palm

**Figure 6.**
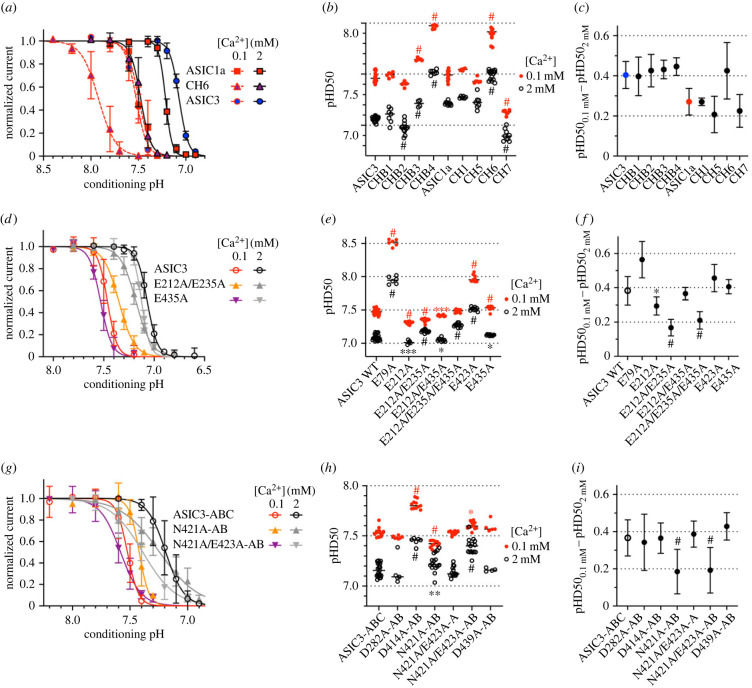
The modulation of SSD by Ca^2+^ depends on residues in the acidic pocket and the lower palm. Data are from *Xenopus* oocytes expressing the indicated ASIC constructs, obtained by two-electrode voltage clamp to −60 mV. (*a–c*) Data from chimeras; (*d–f*) data from ASIC3 with point mutations; (*g–i*) data from concatamers. (*a,d,g*) pH dependence of SSD for the indicated constructs, obtained with conditioning solutions at a Ca^2+^ concentration of 0.1 or 2 mM; *n* = 6–19. (*b,e,h*) Plot of pHD_50_ (pH of half-maximal SSD) values for the indicated constructs, *n* = 4–26. (*c,f,i*) *Δ*pHD_50_ (pHD_50,0.1mM_ - pHD_50,2mM_ Ca^2+^, mean ± s.d.), *n* = 4–26. *, *p* < 0.05; **, *p* < 0.01; ***, *p* < 0.001; ^#^, *p* < 0.0001; different from the corresponding value obtained with the chimera basis (*b*,*c*), ASIC3 WT (*e*,*f*) and ASIC3 WT-ABC (*h*,*i*); (*b,e,h*) as analysed by one-way ANOVA followed by Dunnett's test; (*c,f,i*) based on permutation analysis (see Methods).

The SSD of the chimeras that generated a transient current was measured at two different Ca^2+^ concentrations in the conditioning solutions, 2 mM and 0.1 mM. The pHD_50_ shift induced by the lowering of the Ca^2+^ concentration from 2 to 0.1 mM was 0.27 ± 0.07 with ASIC1a, and 0.40 ± 0.07 with ASIC3 (*n* = 10–24 (figure 6*a–c*). The SSD curves showed some shifts in the pH dependence of SSD (figure 6*b*); however, the ΔpHD_50_ was in all cases except for CH6 close to the parent channel type value (figure 6*c*). Due to the high variability in the CH6 *Δ*pHD50 values, the CH6 ΔpHD50 was not significantly different from that of ASIC1a WT. In the chimera CH6, the thumb and part of the thumb-palm linkers is replaced by the corresponding sequence of ASIC3.

Functional analysis of ASIC3 mutants showed low ΔpHD_50_ values in the mutants E212A, E212A/E235A and E212A/E235A/E435A (figure 6*d–f*). Mutation of the pore residue Glu435 did not affect Ca^2+^ modulation of the SSD. This analysis identifies therefore E212 and E235 as important for SSD. These residues are located in the acidic pocket and are part of the palm and finger, respectively (figure 3*d*). Measurement of concatameric channels showed low *Δ*pHD_50_ values with N421A-AB and N421A/E423A-AB (figure 6*g–i*). Because the mutation E423A induced an alkaline shift of the pHD_50_ at both Ca^2+^ concentrations but did not decrease the ΔpHD_50_ (figure 6*e,f*), the observed decrease of the *Δ*pHD_50_ with N421A/E423A-AB identifies Asn421 as important for the Ca^2+^ modulation of the SSD. Asn421 is located in the lower palm (figure 3*c*). To combine the SSD-affecting mutations in the acidic pocket and wrist, we constructed a concatamer containing the mutations E212A and E235A in all three, and N421A in two subunits. This construct expressed only very small currents that were mostly sustained and did therefore not allow us to determine the Ca^2+^ dependence of SSD. Taken together, the analysis with chimeras and two mutagenic approaches identified Glu residues 212 and 235 of the acidic pocket, and Asn421 of the palm as contributing residues to Ca^2+^ modulation of SSD.

## 3. Discussion

Extracellular Ca^2+^ tunes the pH dependence of ASIC3. We show here that at high Ca^2+^ concentrations, the pH dependence of activation and of SSD is shifted to more acidic values. We identify residues in the pore entry and in the acidic pocket that are critically involved in the regulation of ASIC3 pH dependence of activation by Ca^2+^, and residues of the acidic pocket and the palm that are involved in the modulation by Ca^2+^ of the pH dependence of SSD. Together, our data show that Ca^2+^ binding sites outside and within the pore entry are involved in the control of ASIC3 activity by Ca^2+^.

Basal interstitial Ca^2+^ concentrations in the brain of mammals are between 1 and 2 mM. During high neuronal activity, this concentration decreases to approximately 0.8 mM or lower [1,29,30]. Following ischaemic stroke, a stronger decrease in Ca^2+^ concentration (to approx. 0.1 mM) occurs [2]. A heart attack and muscle stress or ischaemia are generally associated with an increased production of lactate. Since lactate is a weak Ca^2+^ chelator, this results in a lower concentration of free Ca^2+^ [3]. Thus, free extracellular Ca^2+^ concentrations change under some conditions, and since Ca^2+^ and other ligands compete for ion channel binding, this can thereby affect the activity of ASICs and other ion channels.

Lowering of the extracellular Ca^2+^ concentration shifts the voltage dependence of voltage-gated Na^+^ channels to more hyperpolarized potentials [5,31]. It was concluded that this effect was due to reduced surface charge screening at lower Ca^2+^ concentrations [5]. The sodium leak channel (NALCN) is modulated by voltage and by the Ca^2+^ concentration. It is inhibited by extracellular Ca^2+^ with an IC_50_ of 320 µM at −80 mV; this inhibition is likely due to a pore block, since it is strongly suppressed by mutations of selectivity filter residues [6]. The calcium homeostasis modulator 1 (CALHM1) is, similarly to NALCN, regulated by voltage and by the extracellular Ca^2+^ concentration. Reducing the Ca^2+^ concentration increases the channel open probability [7]. The IC_50_ for CALHM1 inhibition by Ca^2+^ is approximately 200 µM at −60 mV. Neutralization of an extracellular Asp residue that is not in the permeation pathway was shown to strongly alter the regulation by Ca^2+^ [7].

**Figure 7.**
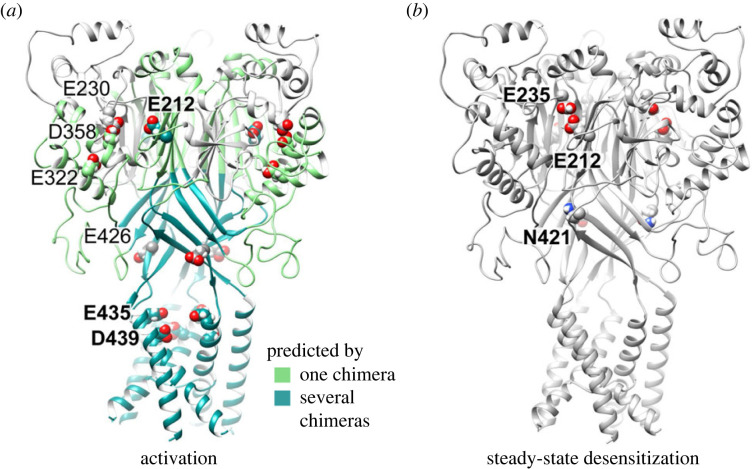
Domains and residues involved in the Ca^2+^ modulation of activation and of SSD. The images were made from a homology model of ASIC3, based on the crystal structure of the open chicken ASIC1a, 4NTW [17]. (*a*) Domains that were shown to be involved in Ca^2+^ modulation of activation with one chimera are coloured in light green, those shown in more than one chimera in dark green; the residues identified with mutants are indicated. Residues whose mutation induced the strongest changes are labelled in bold. (*b*) Residues that were shown with mutants to be involved in Ca^2+^ modulation of SSD are indicated.

Our mutagenesis approach showed that the most important residues for Ca^2+^ modulation of ASIC3 activation are located in the acidic pocket and the pore entry (figure 7*a*). This is consistent with the analysis of chimeras indicating a contribution of the transmembrane segments, the thumb and the palm. Mutation of Glu435 in ASIC3 suppressed only partially the low Ca^2+^-induced current at pH7.4 in the study by Zuo *et al*. [21] and in our hands. The E435A and E212A mutations inhibited the low Ca^2+^-induced shift in the activation curve to a similar degree. In concatamers, the presence of the D439A mutation in two of three subunits inhibited the Ca^2+^ modulation to a higher degree than the single E212A and E435A mutation in the three subunits (i.e. in conventional mutants), underlining the importance of Asp439 for Ca^2+^ modulation of ASIC3. Of these three residues, both Glu212 and Asp439 are conserved between ASIC3 and ASIC1a; only Glu435 is unique to ASIC3. The different roles of these identical residues in ASIC1a and ASIC3 is therefore likely due to (i) differences in the amino acid residues or conformation in the proximity of these residues that may affect the Ca^2+^ affinity and/or (ii) a different coupling of these Ca^2+^ binding sites to ASIC activity.

The mutations that decreased the ΔpH_50_ did this mostly by lowering the pH_50_ at the 80 nM Ca^2+^ condition, and with no, or only minor changes of the pH_50_ at 2 mM Ca^2+^. Since these mutations disrupt the binding of Ca^2+^, it would seem more logical that they have a stronger effect at the higher Ca^2+^ concentration. As a possible explanation for this apparent discrepancy, we hypothesize that the charge of the acidic side chains at the Ca^2+^ binding sites may allow the high pH sensitivity in the absence of Ca^2+^. If such acidic side chains are removed by mutagenesis, the channels may lose the possibility of gating with high pH sensitivity.

The analysis of SSD showed that the acidic pocket residues Glu212 and Glu235 are important for the competition between Ca^2+^ and H^+^ (figure 7*b*). The SSD analysis further highlighted the contribution of Asn421 located in the lower palm, which may form a Ca^2+^ binding site together with Glu79 and Glu423. These residues are also conserved between ASIC3 and ASIC1a.

For ASIC1a, it was shown that mutation of the two residues in the ASIC1a pore that contribute to Ca^2+^ block, corresponding to ASIC3-Glu432 and -Asp439, suppressed the pore block but not the Ca^2+^ modulation of the pH dependence, indicating that the Ca^2+^ binding site for gating is different from that for pore block in ASIC1a [23]. A study with toadfish ASIC1 also found a competition between H^+^ and Ca^2+^ and concluded, based on a detailed functional analysis, that this channel is activated by an allosteric mechanism and not by a release of Ca^2+^ block [32]. A further indication that in ASIC1a, activation involves conformational changes comes from voltage clamp fluorometry studies that detected conformational changes associated with channel opening [33,34].

At the homologous position to ASIC3-Glu435, ASIC1a contains a Gly residue (Gly430). When the homologous Gly residue in chicken ASIC1a (cASIC1a) was mutated to Glu—in order to create a site favourable for Ca^2+^ binding—the shift of the activation curve induced by lowering of the Ca^2+^ concentration was increased relative to that seen in WT cASIC1a [21]. In this mutant, the removal of extracellular Ca^2+^ activated the channel at pH7.4 [21], indicating therefore that an acidic side chain at this position of ASIC1a has a similar role as does Glu435 in ASIC3. A recent study showed that if ASIC1a-Gly430 was mutated to Cys, exposure to Cys-reactive reagents shifted the pH dependence of activation towards alkaline values, indicating that large side chains at this position facilitate ASIC1a opening [35] in a similar way as lowering the Ca^2+^ concentration shifts the pH dependence of ASIC3.

The original study describing the competition between H^+^ and Ca^2+^ for the activation of ASIC3 concluded that this competition occurs in the pore entry, based on the good fit by a kinetic model of the H^+^ and Ca^2+^ dependence of the currents, and the dependence of the unitary current amplitude on the Ca^2+^ concentration [8]. It was proposed that ASIC3 opening is due to unbinding of Ca^2+^ bound to the pore entry, without any conformational changes involved. This conclusion was later supported by the demonstration of the involvement of Glu435 in the Ca^2+^ modulation [21]. A structural analysis of closed and desensitized ASIC1a identified divalent ion binding sites in the acidic pocket and the central vestibule, but not in the pore [26]. In the structural model of closed hASIC1a, the O–O distances of the D439 side chains around the pore were approximately 4 Å [19], thus compatible with a Ca^2+^ binding site [36,37]. In binding sites containing multiple coordinating ligands, Ca^2+^ can bind with reasonable affinity even if it is hydrated. In such cases, the ion is placed 4–5 Å from the interacting side chains [38]. According to the original hypothesized mechanism [8], Ca^2+^ binds into the open ASIC3 pore. The ASIC3 structural model is based on the chicken ASIC1a structure [17]. ASIC1a and ASIC3 have similar Na^+^/K^+^ permeability ratios, suggesting that at least at the selectivity filter they have similar pore radii. Assuming that the pore dimensions are correct in the ASIC3 open channel model, the three Asp439 side chains would not be able to coordinate binding of a Ca^2+^ ion between them, because of side chain O–O distances of greater than 12 Å. At the level of Glu435, the intersubunit carboxylic O–O distances are also close to 12 Å. The closest distance between side chain oxygens of Glu435 and Asp439 of the same subunit is 6.9 Å; therefore, it seems also unlikely that Glu435 and Asp439 would together coordinate a Ca^2+^ ion in the open ASIC3 pore. This indicates that in a pore conformation compatible with ion flow through the ASIC3 pore, the Glu435 or Asp439 residues of the three subunits cannot coordinate a Ca^2+^ ion, strongly suggesting that conformational changes in the pore accompany ASIC3 activation.

Based on our functional analysis and the comparison with the ASIC1a open pore dimensions, we conclude that ASIC3 activity is controlled by a hybrid mechanism involving on one hand conformational changes induced by protonation of an allosteric site, and on the other hand a release of Ca^2+^ bound to the pore entry. At binding sites distant from the pore, Ca^2+^ interferes with allosteric gating by competing with H^+^. The competition for binding sites in the pore leads to release of Ca^2+^ from the pore when the pH is lowered.

## 4. Methods

### 4.1. Molecular biology

The rat ASIC3 clone [39] and the human ASIC1a clone [40] and derived mutants and chimeras were subcloned into a pSP65-derived vector containing 5′ and 3′ non-translated sequences of *β* globin to improve the stability in *Xenopus* laevis oocytes. The ASIC1a clone used in this study contains a G212D substitution [41]. Mutations were generated by site-directed mutagenesis using the Quikchange approach, with KAPA HiFi HotStart PCR polymerase (KAPA Biosystems). Mutations were verified by sequencing (Synergen Biotech or Microsynth). Chimeras between ASIC1a and ASIC3 were designed based on a sequence alignment and the attribution of subdomains based on [16] and were synthesized by Genscript or Eurofins. The protein sequences of these constructs are provided in the electronic supplementary material, table S1. The concatamer constructs contained three repeats of ASIC3 subunits, A, B and C. These repeats contained the mutation L529A that had been shown to increase the current by approximately threefold without changing other functional properties of the channel [42]. The linker between repeats A and B was composed of the sequence QQQASQQ, while the linker between repeats B and C contained the sequence NNNNTSNNN. The presence of a HindIII and a SpeI restriction sites in the A-B and B-C linkers, respectively, allowed the subcloning of mutant repeats at the desired positions. The concatameric constructs were cloned in the oocyte expression vector psDeasyBS with the EcoRI and XbaI sites. The concatamers were synthesized in part or completely by Genscript. The complete coding sequence of chimeras and concatamers was verified by sequencing (Synergen Biotech or Microsynth).

### 4.2. Oocytes and electrophysiological measurements

Female *Xenopus laevis* frogs were anaesthetized with 1.3 g L^−1^ MS-222 (Sigma-Aldrich). For the extraction of oocytes, a small incision (approx. 1 cm) was made on the abdominal wall. The procedures with the *Xenopus laevis* frogs were approved by the veterinary office of the canton de Vaud. Healthy stage V and VI oocytes were isolated and incubated with collagenase to isolate and defolliculate the oocytes. Oocytes were injected with 50 nL of cRNA at a concentration of 20–500 ng µL^−1^. During the protein expression phase, the oocytes were stored in modified Barth's saline containing (in mM) 85 NaCl, 1 KCl, 2.4 NaHCO_3_, 0.33 Ca (NO_3_)_2_, 0.82 MgSO_4_, 0.41 CaCl_2_, 10 HEPES and 4.08 NaOH at 19°C.

It has been reported that *Xenopus* oocytes express endogenous connexin hemichannels, and that lowering of the extracellular Ca^2+^ concentration can induce inward currents [22]. Control experiments with non-injected oocytes showed that switching at pH7.4 from a solution containing 2 mM Ca^2+^ to a solution containing 100 nM free Ca^2+^ induced a slowly developing inward current that still increased at the end of the 10 s solution change (electronic supplementary material, figure S1A). Ca^2+^ was previously shown to inhibit these endogenous currents with an IC_50_ of approximately 100 µM at −60 mV [43]; therefore, the inhibition is not different between the 100 nM used in these control experiments and the 80 nM used with ASIC3.

Interestingly, the amplitude of the endogenous currents was smaller at pH6 and was inhibited with a pH_50_ of 5.41 ± 0.41 (*n* = 6; electronic supplementary material, figure S1A,B). The peak of ASIC currents appears normally within the first second after solution change (see e.g. figure 1*b*). The amplitude of the current induced in non-injected oocytes by lowering of the Ca^2+^ concentration to 100 nM at pH7.4, measured at 3.5 s after the solution change (which would be after the ASIC peak in ASIC-expressing oocytes) was −93 ± 50 nA (*n* = 29, from three different batches of oocytes; electronic supplementary material, figure S1C). To estimate the consequences of the presence of endogenous low Ca^2+^-activated currents for the analysis of the ASIC3 pH dependence, pH dependence curves were generated *in silico* with Hill equations describing the pH dependence of the ASIC3 WT and of endogenous low Ca^2+^-activated currents (*I* = *I*_max_/(1+(10^−pH^_50_/10^−pH^)^nH^), where *I*_max_ is the maximal current amplitude, pH_50_ is the pH inducing 50% of the maximal current amplitude and nH is the Hill coefficient). For ASIC3 WT, the pH_50_ and nH were 7.48 and 2.08, respectively, as determined in figure 1*d*. For the endogenous currents, the fit parameters determined in the electronic supplementary material, figure S1B were used. Activation curves were generated for pure ASIC3 and for conditions in which the endogenous current contributed 5, 10, 15 or 20% to the total maximal peak current amplitude (electronic supplementary material, figure S1D,E). As expected from the opposite pH dependence of the two current types, contamination by endogenous currents affected the resulting pH dependence mostly at pH > 7.3). An endogenous current amplitude corresponding to 10% of the maximal total peak current induced for example an alkaline shift of the pH_50_ value of 0.07 pH units and a decrease of nH from 2.08 to 1.57. With the measured amplitude of the endogenous current of 93 nA, such an error would be expected for ASIC currents with a maximal peak amplitude of 0.93 µA. Of the ASIC constructs used in this study, the maximal peak current in activation curves with 80 nM Ca^2+^ was 0.92 µA for the chimera CHB6, 1.84 µA in the concatamer N421A/E423A-AB, and greater than 2 µA for all other constructs (electronic supplementary material, table S2), indicating therefore that the endogenous currents did not affect the measurement of the ASIC pH dependence. The current traces of experiments with low ASIC current amplitudes at 80 nM Ca^2+^ were visually inspected and were only used if at pH conditions of greater than or equal to 7.3, the amplitude of the slowly activating endogenous current was less than 5% of the maximal low pH-induced current amplitude. For SSD experiments, the conditioning solution contained either 2 mM or 0.1 mM Ca^2+^. If at pH7.4, the Ca^2+^ concentration was lowered during 10 s to 0.1 mM, the current amplitude relative to the baseline at pH7.4/2 mM Ca^2+^ was 15 ± 9 nA at pH7.4 and 12 ± 8 nA at pH8.5, thus negligeable (*n* = 8, electronic supplementary material, figure S1F).

Electrophysiological recordings were performed 1–3 days after cRNA injection as described [44]. The currents were recorded using the two-electrode voltage clamp technique at a holding potential of −60 mV unless noted differently. Recordings were carried out with a Dagan TEV200 amplifier (Minneapolis, MN) equipped with two bath electrodes, using either the Clampex 9.2 (Molecular Devices) or the ChartMaster software (HEKA Elektronik-Harvard Bioscience), and for analysis the Clampfit or FitMaster software. The recording solution contained (in mM): 110 NaCl, 10 HEPES for pH ≥ pH6.8 (MES for pH < 6.8) and the appropriate Ca^2+^ concentration. The pH was adjusted using NaOH or HCl. For Ca^2+^ concentrations greater than or equal to 0.1 mM, the Ca^2+^ was provided as CaCl_2_, and no Ca^2+^ chelator was added. For Ca^2+^ concentrations less than 0.1 mM, Ca^2+^ chelators at a concentration of generally 10 mM were used and total Ca^2+^ concentrations were chosen based on the MaxChelator program (https://somapp.ucdmc.ucdavis.edu/pharmacology/bers/maxchelator/webmaxc/webmaxcS.htm [45]) to obtain the desired free Ca^2+^ concentration. Depending on the pH and the free Ca^2+^ concentration, EDTA, EGTA or citrate were used as chelators. Once placed in the recording chamber, the oocyte was impaled with two glass electrodes that had a resistance of less than 0.5 MΩ when filled with 1 M KCl. Oocytes were perfused with experimental solutions by gravity at a flow rate of 8–12 mL min^−1^. The cFlow 8 channel flow controller (CellMicroControls) together with an eightfold perfusion head was used to change solutions. Currents were filtered at 2 kHz and the sampling interval was 20 ms. The oocytes were exposed once per minute for 5–15 s (according to the specific protocol used) to the stimulation solution. The pH activation curves were fit to the Hill equation that is described above. An analogous equation was used to fit the SSD curves.

### 4.3. Computational analysis

The structural model of ASIC3 used here was created with SWISS-MODEL [46] based on the open chicken ASIC1 structure (4NTW [17]). Molecular simulation systems were composed of an ASIC3 protein trimer surrounded by about 360 POPC lipid molecules forming a bilayer. This protein/membrane complex is solvated by about 36 000 water molecules and 100 mM of Ca^2+^, to which Na^+^ and Cl^−^ ions are added to neutralize the whole system and mimic a salt concentration of about 150 mM. Following an approach developed by Woolf & Roux [47], simulation systems were first equilibrated with a set of gradually decreasing restraints using the CHARMM software. Molecular dynamics simulations were then performed with the NAMD package [48].

As we were interested in potential Ca^2+^ binding sites which are in competition with protonation of side chains, we ran two simulations with all acidic side chains deprotonated (all histidines were also neutral) for a total of 520 ns of simulation time. One of these two simulations additionally had a transmembrane potential of 200 mV to favour the entry of Ca^2+^ ions into the pore to identify potential residues involved in Ca-dependent pore block. A third simulation was run for 200 ns with protonation states of Glu and Asp residues adjusted to mimic pH5.5, thus with side chains with pKa ≥ 5.5 deprotonated; the pKa values were estimated by propKa [49,50]. In this system, residues D78, E79, E221, E231, E235, E247, E322, D351, D358, D414, E418, E423 and D439 were protonated and all His residues were neutral. Results with and without transmembrane potential were similar and are aggregated for clarity.

### 4.4. Quantification and statistical analysis

The fits and the statistical analyses were carried out with Graphpad Prism, Version 9. Statistical differences between two groups were analysed with student's *t*-test and differences between greater than two groups were analysed with ANOVA (or Kruskal–Wallis test for not normally distributed data) followed by a Dunnett's *post hoc* test. Data are presented as mean ± s.d. or as individual data points. The significance of the difference of *Δ*pH_50_ or *Δ*pHD_50_ values between mutants and WT channels was assessed using a permutation test, with *n* = 10 000 permutations, using the R statistical language (version 4.2.1).

## Data Availability

This paper does not report original code. All data supporting the findings of this study are available within the main manuscript and the electronic supplementary material [51].
